# Crystal structure and Hirshfeld surface analysis of 1-(*tert*-butyl­amino)-3-mesitylpropan-2-ol hemi­hydrate

**DOI:** 10.1107/S2056989022004297

**Published:** 2022-04-28

**Authors:** Ali N. Khalilov, Victor N. Khrustalev, Tatiana A. Tereshina, Mehmet Akkurt, Rovnag M. Rzayev, Anzurat A. Akobirshoeva, İbrahim G. Mamedov

**Affiliations:** a "Composite Materials" Scientific Research Center, Azerbaijan State Economic University (UNEC), H. Aliyev str. 135, Az 1063, Baku, Azerbaijan; b Department of Chemistry, Baku State University, Z. Khalilov str. 23, Az 1148, Baku, Azerbaijan; c Peoples’ Friendship University of Russia (RUDN University), Miklukho-Maklay St. 6, Moscow, 117198, Russian Federation; d N. D. Zelinsky Institute of Organic Chemistry RAS, Leninsky Prosp. 47, Moscow, 119991, Russian Federation; e Department of Physics, Faculty of Sciences, Erciyes University, 38039 Kayseri, Turkey; f Acad. Sci. Republ. Tadzhikistan, Kh. Yu. Yusufbekov Pamir Biol Inst, 1 Kholdorova St, Khorog 736002, Gbao, Tajikistan

**Keywords:** crystal structure, 1,2-amino alcohols, hydrogen bonds, C—H⋯π inter­actions, Hirshfeld surface analysis

## Abstract

The title compound, 2C_16_H_27_NO·H_2_O, crystallizes in the monoclinic *P*2_1_/*c* space group with two independent mol­ecules (*A* and *B*) in the asymmetric unit. In the crystal, mol­ecules *A* and *B* are linked through the water mol­ecules by inter­molecular O—H⋯O and O—H⋯N hydrogen bonds, producing chains along the *b*-axis direction. These chains are linked with neighboring chains parallel to the (103) plane *via* C—H⋯π inter­actions, generating ribbons along the *b*-axis direction. The stability of the mol­ecular packaging is ensured by van der Waals inter­actions between the ribbons. According to the Hirshfeld surface study, H⋯H inter­actions are the most significant contributors to the crystal packing (80.3% for mol­ecule *A* and 84.8% for mol­ecule *B*).

## Abstract 2

In the crystal, the two independent mol­ecules are linked through the water mol­ecules by inter­molecular O—H⋯O and O—H⋯N hydrogen bonds, producing chains along the *b*-axis direction. These chains are linked with neighboring chains parallel to the (103) plane *via* C—H inter­actions, generating ribbons along the *b* axis. The van der Waals inter­actions between the strips ensure the stability of mol­ecular packaging.

## 1. Chemical context

Amine group-containing compounds are of great inter­est in the fields of organic synthesis, catalysis, material science and medicinal chemistry (Zubkov *et al.*, 2018 ▸; Shikhaliyev *et al.*, 2019 ▸; Viswanathan *et al.*, 2019 ▸; Gurbanov *et al.*, 2020 ▸). In particular, the β-amino alcohol moiety is the predominant structural motif in a series of natural and synthetic biologically active mol­ecules (Lee & Kang, 2004 ▸). Amino alcohol derivatives are currently being studied for their anti­microbial, anti­fungal, anti­oxidant, cytotoxic, enzyme inhibitory and other important biological activities, which have been well documented in recent works (Baker *et al.*, 2021 ▸; Estolano-Cobián *et al.*, 2020 ▸; Tafelska-Kaczmarek *et al.*, 2020 ▸). 
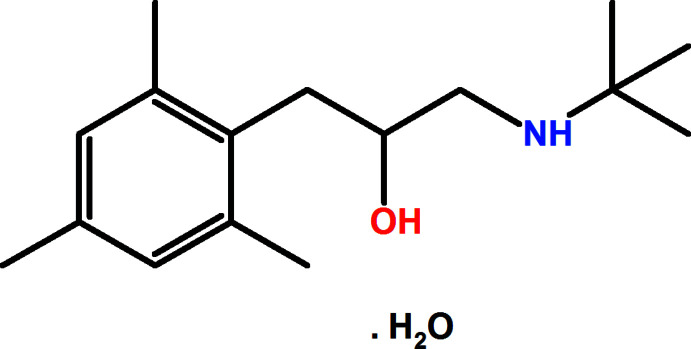


In this study, in the framework of ongoing structural studies (Safavora *et al.*, 2019 ▸; Aliyeva *et al.*, 2011 ▸; Mamedov *et al.*, 2022 ▸), we report the crystal structure and Hirshfeld surface analysis of the title compound, 1-(*tert*-butyl­amino)-3-mesitylpropan-2-ol hemihydrate.

## 2. Structural commentary

**Figure 1:**
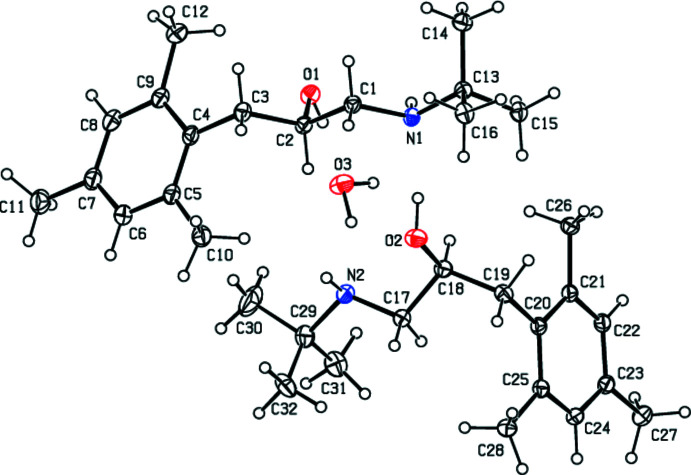
View of the two independent mol­ecules, *A* and *B*, in the asymmetric unit of the title compound, with displacement ellipsoids for the non-hydrogen atoms drawn at the 30% probability level. For clarity, the minor components of disorder in mol­ecule *B* are omitted.

**Figure 2:**
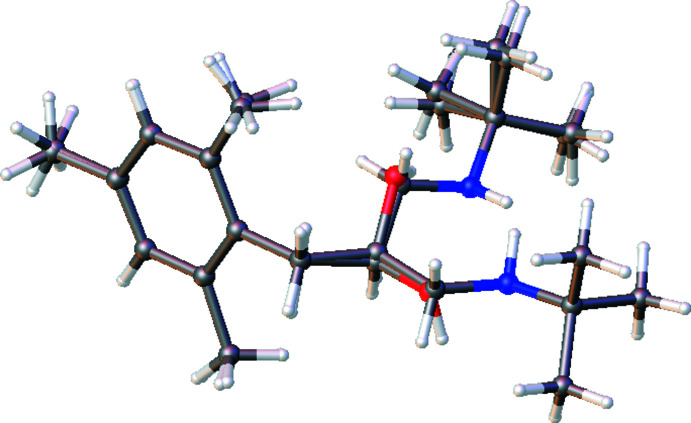
Overlay image of the two independent mol­ecules (*A* and *B*) in the asymmetric unit of the title compound. Both the major and minor components of disorder in mol­ecule *B* are shown. Color code: carbon (gray), hydrogen (white), nitro­gen (blue) and oxygen (red).

The title compound (Fig. 1 ▸) contains the two independent mol­ecules (mol­ecule *A* containing atom N1 and mol­ecule *B* containing N2) in the asymmetric unit. As shown in Fig. 2 ▸ (r.m.s. deviation = 0.006 Å), while the 1,2,3,5-tetra­methyl­benzene parts of mol­ecules *A* and *B* are overlapped, their 2-(*tert*-butyl­amino)­ethan-1-ol moieties do not overlap, but rather are oriented in opposite directions. Atoms C2 in mol­ecule *A* and C18 in mol­ecule *B* have opposite chiralities. The chirality about the C2 atom is *R* and that about C18, *S*. The values of the geometric parameters of mol­ecules *A* and *B* are normal and compatible with those of the related compounds mentioned in the *Database survey* section.

## 3. Supra­molecular features and Hirshfeld surface analysis

**Figure 3:**
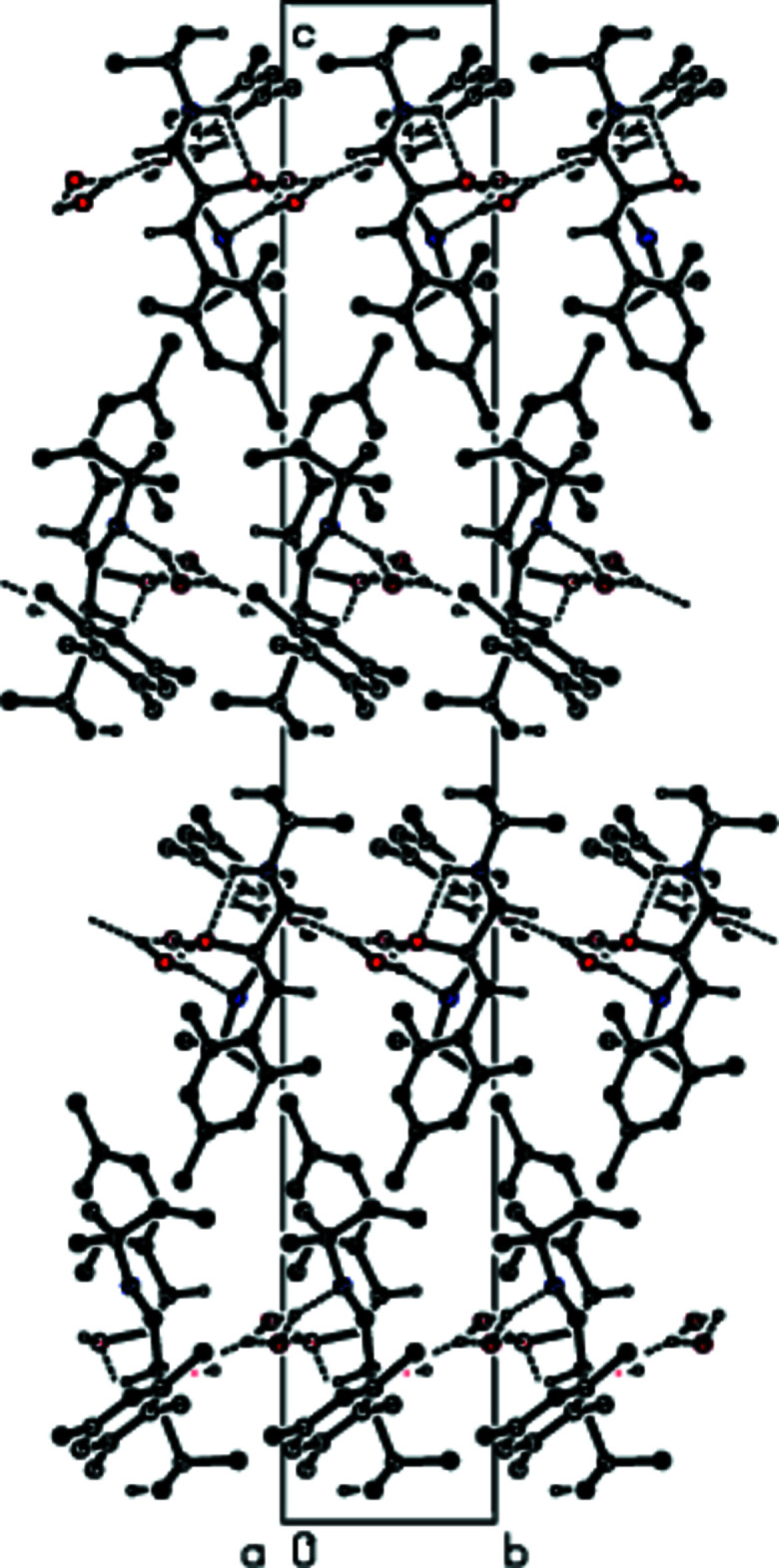
A view of the inter­molecular O—H⋯O and O—H⋯N inter­actions along the *a* axis in the crystal structure of the title compound. For clarity, H atoms not involved in hydrogen bonding and the minor disorder components in mol­ecule ***B*** are omitted.

**Figure 5:**
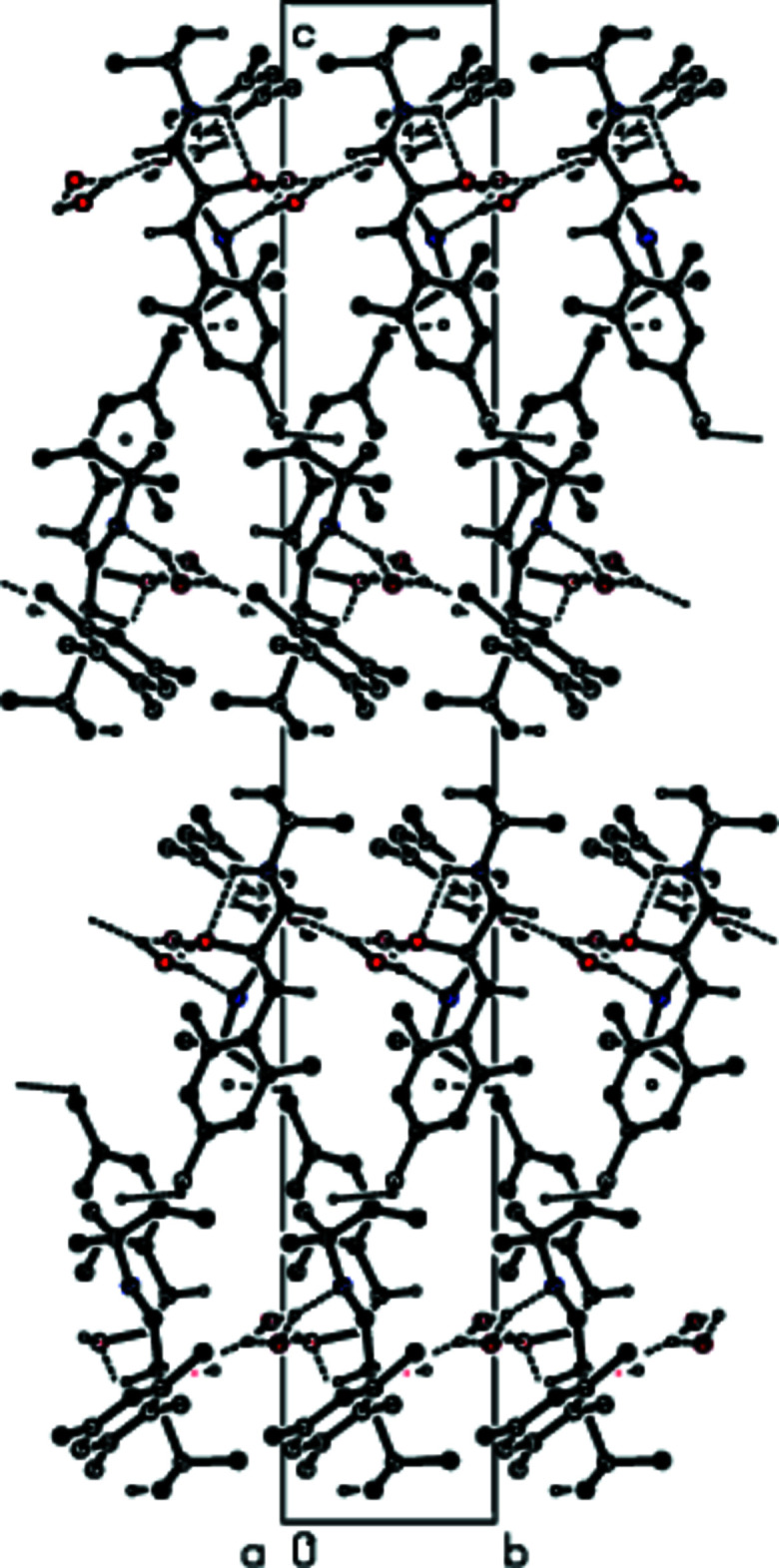
A view of the inter­molecular O—H⋯O and O—H⋯N inter­actions and C—H⋯π inter­actions along the *a* axis in the crystal structure of the title compound. For clarity, H atoms not involved in hydrogen bonding and the minor disorder components in mol­ecule ***B*** are omitted.

In the crystal, mol­ecules *A* and *B* are linked through the water mol­ecules by inter­molecular O—H⋯O and O—H⋯N hydrogen bonds (Table 1 ▸; Figs. 3 ▸ and 4 ▸), forming chains along the *b*-axis direction. These chains are linked by C—H⋯π inter­actions with neighboring chains parallel to the (103) plane, forming ribbons along the *b*-axis direction (Table 1 ▸; Figs. 5 ▸ and 6 ▸). The stability of the mol­ecular packing is ensured by van der Waals inter­actions between the ribbons.

**Figure 7:**
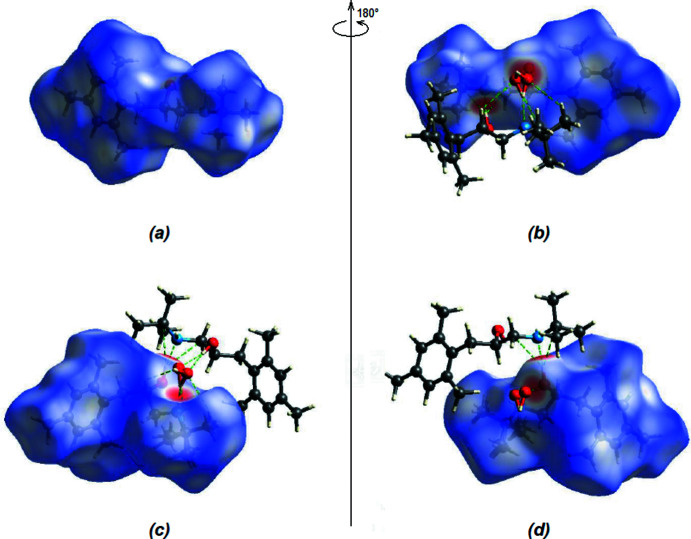
Front (*a*) and back (*b*) views of the three-dimensional Hirshfeld surface for mol­ecule *A*. Front (*c*) and back (*d*) views of the three-dimensional Hirshfeld surface for mol­ecule *B*. Some inter­molecular O—H⋯O and O—H⋯N inter­actions are shown.

Hirshfeld surfaces were generated for both independent mol­ecules using *Crystal Explorer 17* (Turner *et al.*, 2017 ▸). The *d*_norm_ mappings for mol­ecules *A* and *B* were performed in the ranges −0.6784 to 1.2952 a.u. and −0.6765 to 1.3828 a.u., respectively. The O—H⋯O and O—H⋯N inter­actions are indicated by red areas on the Hirshfeld surfaces (Fig. 7 ▸ *a*,*b* for *A* and Fig. 7 ▸ *c*,*d* for *B*).

**Figure 8:**
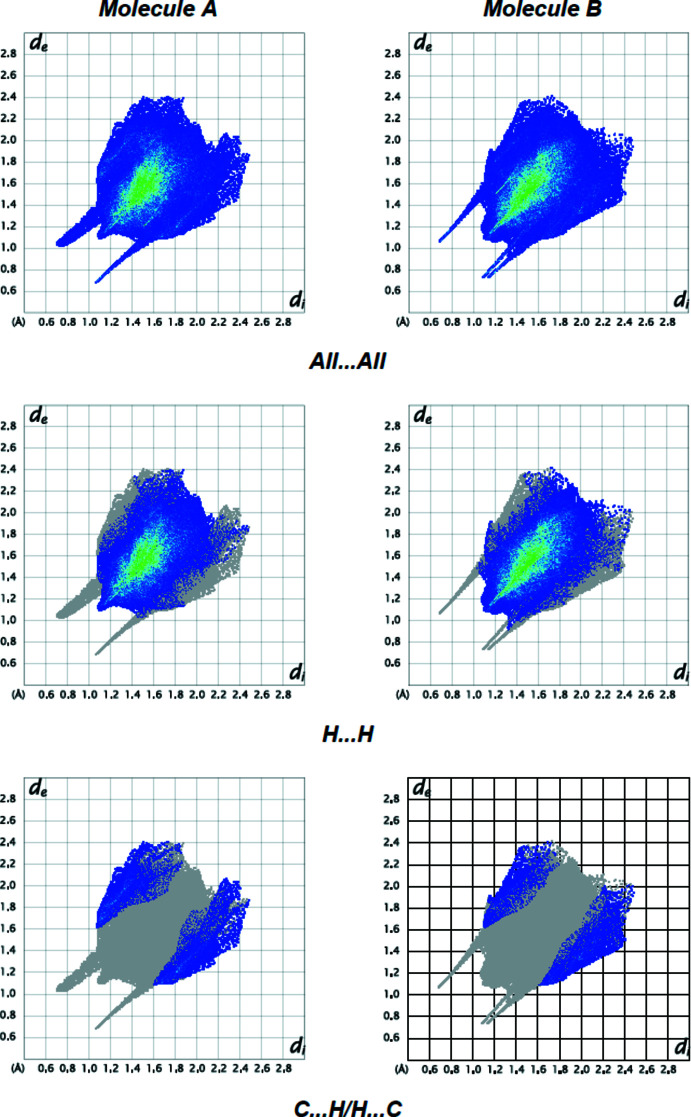
The two-dimensional fingerprint plots for mol­ecules *A* and *B* of the title compound showing (*a*) all inter­actions, and delineated into (*b*) H⋯H and (*c*) C⋯H/H⋯C inter­actions. The *d*_ĩ_ and *d*_e_ values are the closest inter­nal and external distances (in Å) from given points on the Hirshfeld surface.

Fingerprint plots (Fig. 8 ▸) reveal that while H⋯H inter­actions (80.3% for mol­ecule *A* and 84.8% for mol­ecule *B*) make the largest contributions to surface contacts (Tables 1 ▸ and 2 ▸), C⋯H/H⋯C contacts (13.0% for mol­ecule *A* and 9.1% for mol­ecule *B*) are also important. Other, less notable linkages are O⋯H/H⋯O (5.7% contribution for mol­ecule *A* and 4.3% for mol­ecule *B*) and N⋯H/H⋯N (1.0% for mol­ecule *A* and 1.8% for mol­ecule *B*). The surroundings of mol­ecules *A* and *B* are very similar, as can be observed from the comparison of the supplied data.

## 4. Database survey

Two related compounds were found in a search of the Cambridge Structural Database (CSD, version 5.42, update of September 2021; Groom *et al.*, 2016 ▸), *viz*. 1-methyl­amino-3-(2,4,6-tri­methyl­phen­yl)propan-2-ol (ULIMUY; Maharramov *et al.*, 2011*a* ▸) and 3-[2-hy­droxy-3-(2,4,6-tri­methyl­phen­yl)prop­yl]-3-methyl-1-phenyl­thio­urea (URAPOT; Maharramov *et al.*, 2011*b* ▸).

In ULIMUY, the methyl­amino­propyl chain adopts an extended zigzag conformation and the N atom shows a trigonal coordination. The N atom acts as hydrogen-bond acceptor to the hy­droxy group of an adjacent mol­ecule to generate a helical chain running along the *b*-axis of the monoclinic unit cell.

In URAPOT, the four-atoms N—C(=S)—N unit is planar (r.m.s. deviation of 0.005 Å); the phenyl ring connected to one of the two flanking N atoms is twisted out of this plane by 28.6 (1)°. The propyl chain connected to the other N atom bears a hy­droxy substituent; this serves as hydrogen-bond donor and acceptor to the double-bonded S atom of an inversion-related mol­ecule, generating a hydrogen-bonded dimer.

## 5. Synthesis and crystallization

The title compound was synthesized using our previously reported procedure (Khalilov *et al.*, 2021 ▸), and colorless crystals were obtained upon recrystallization from an ethanol solution.

## 6. Refinement

Crystal data, data collection and structure refinement details are summarized in Table 3 ▸. Carbon-bound H atoms were placed in calculated positions [C—H = 0.95 to 1.00 Å; *U*_iso_(H) = 1.2 or 1.5*U*_eq_(C)] and were included in the refinement in the riding-model approximation. The hy­droxy and amino H atoms were located in a difference Fourier map, and were freely refined [O1—H1*O* = 0.91 (2) Å, O2—H2*O* = 0.91 (2) Å, N1—H1*N* = 0.922 (16) Å and N2—H2*N* = 0.922 (18) Å]. In mol­ecule *B*, the methyl groups of the 2-methyl­propane moiety are disordered over two sets of sites with an occupancy ratio of 0.65 (3):0.35 (3). The water mol­ecule is disordered over two positions with an occupancy ratio of 0.59 (3):0.41 (3). The two H atoms of the water mol­ecule were found in a difference-Fourier map and freely refined [O3—H3*C* = 0.95 (2) Å, O3—H3*D* = 0.98 (3) Å, O3′—H3*C* = 0.92 (2) Å and O3′—H3*D* = 1.07 (3) Å]. The anisotropic displacement parameters of the O3 and O3′ atoms of the disordered water mol­ecule were restrained to be equal (SIMU). SADI and DFIX commands were used for the treatment of the disordered methyl groups of the 2-methyl­propane moiety of mol­ecule *B*.

## Supplementary Material

Crystal structure: contains datablock(s) I. DOI: 10.1107/S2056989022004297/jy2019sup1.cif

Structure factors: contains datablock(s) I. DOI: 10.1107/S2056989022004297/jy2019Isup2.hkl

Supporting information file. DOI: 10.1107/S2056989022004297/jy2019Isup3.cml

CCDC reference: 2168161

Additional supporting information:  crystallographic information; 3D view; checkCIF report

## Figures and Tables

**Figure 4:**
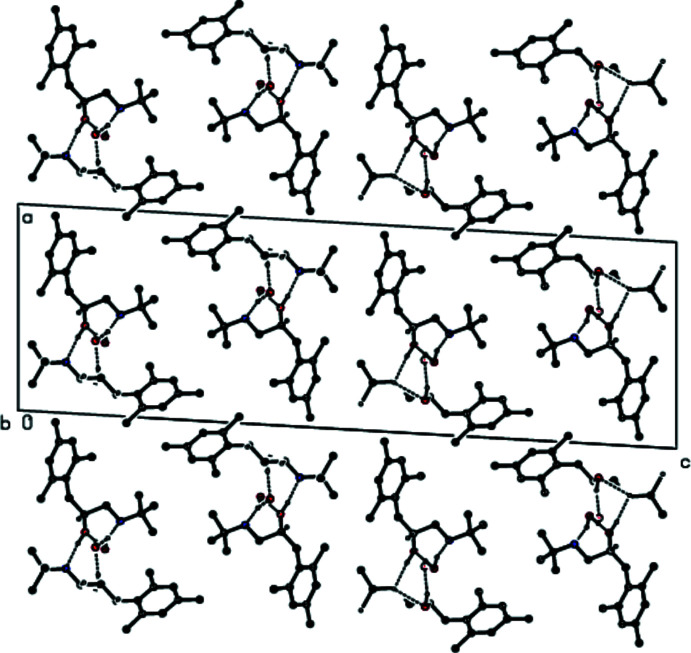
A view of the inter­molecular O—H⋯O and O—H⋯N inter­actions along the *b* axis in the crystal structure of the title compound. For clarity, H atoms not involved in hydrogen bonding and the minor disorder components in mol­ecule ***B*** are omitted.

**Figure 6:**
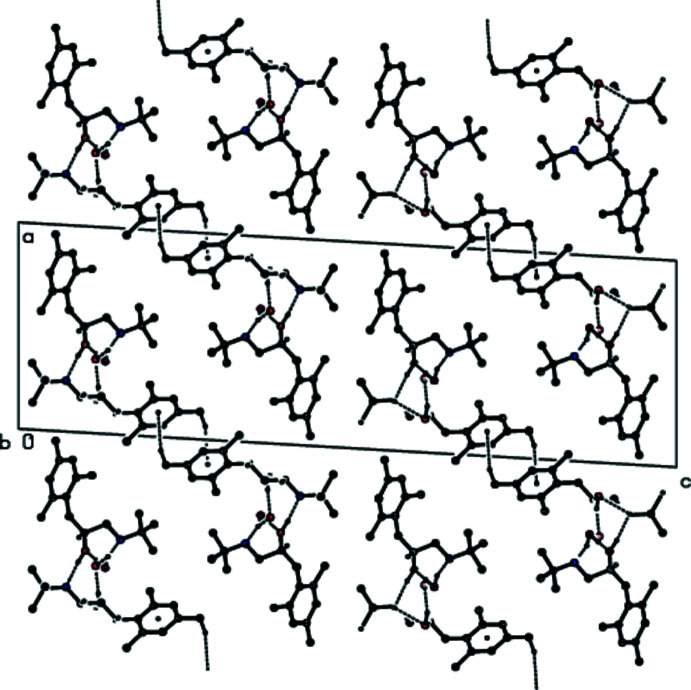
A view of the inter­molecular O—H⋯O and O—H⋯N inter­actions and C—H⋯π inter­actions along the *b* axis in the crystal structure of the title compound. For clarity, H atoms not involved in hydrogen bonding and the minor disorder components in mol­ecule ***B*** are omitted.

**Table 1:** Hydrogen-bond geometry (Å, °) *Cg*2 is the centroid of the benzene ring (C4–C9) of mol­ecule *A*.

*D*—H⋯*A*	*D*—H	H⋯*A*	*D*⋯*A*	*D*—H⋯*A*
O1—H1*O*⋯O3	0.91 (2)	1.82 (2)	2.725 (5)	173 (2)
O1—H1*O*⋯O3′	0.91 (2)	1.82 (2)	2.697 (6)	161 (2)
O2—H2*O*⋯N1	0.91 (2)	1.83 (2)	2.7273 (13)	168.0 (19)
O3—H3*C*⋯O2^i^	0.95 (2)	1.83 (2)	2.753 (3)	162 (2)
O3′—H3*C*⋯O2^i^	0.92 (2)	1.83 (2)	2.685 (4)	153 (2)
O3—H3*D*⋯N2	0.98 (3)	1.87 (3)	2.827 (3)	164 (2)
O3′—H3*D*⋯N2	1.07 (3)	1.87 (3)	2.875 (5)	155 (2)
C11—H11*B*⋯*Cg*2^ii^	0.98	2.90	3.7613 (17)	147

Symmetry codes: (i) 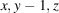 ; (ii) 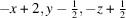 .

**Table 2:** Summary of short inter­atomic contacts (Å) in the title compound

Contact	Distance	Symmetry operation
O2⋯H3*C*	1.83	*x*, 1 + *y*, *z*
H2*O*⋯N1	1.83	*x*, *y*, *z*
N2⋯H3*D*	1.87	*x*, *y*, *z*
H26*C*⋯H15*B*	2.58	1 − *x*, 2 − *y*, 1 − *z*
*H31*D*⋯H17*B*	2.34	*x*, − 1 + *y*, *z*
*H32*B*⋯*H30*E*	2.50	1 − *x*, 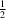 + *y*, 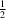 − *z*
H24⋯H3*B*	2.39	−1 + *x*, *y*, *z*
H26*B*⋯H15*C*	2.58	1 − *x*, 1 − *y*, 1 − *z*
*H30*C*⋯C10	3.00	*x*, −1 + *y*, *z*
*H31*B*⋯H6	2.44	1 − *x*, − 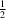 + *y*, 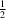 − *z*
*H32*D*⋯H11*C*	2.48	1 − *x*, 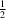 + *y*, 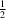 − *z*
H1*O*⋯*O3′	1.82	*x*, *y*, *z*
H1*O*⋯H1*B*	2.46	*x*, −1 + *y*, *z*
C9⋯H11*B*	2.84	2 − *x*, 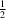 + *y*, 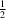 − *z*
H16*C*⋯*O3′	2.89	*x*, 1 + *y*, *z*

The prefix * denotes atoms of the disordered parts of the mol­ecules.

**Table 3:** Experimental details

Crystal data	
Chemical formula	2C_16_H_27_NO·H_2_O
*M*_r_	516.79
Crystal system, space group	Monoclinic, *P*2_1_/*c*
Temperature (K)	100
*a*, *b*, *c* (Å)	13.06508 (16), 5.81242 (6), 41.7384 (5)
β (°)	93.3315 (11)
*V* (Å^3^)	3164.25 (6)
*Z*	4
Radiation type	Cu *K*α
μ (mm^−1^)	0.53
Crystal size (mm)	0.36 × 0.12 × 0.06

Data collection	
Diffractometer	XtaLAB Synergy, Dualflex, HyPix
Absorption correction	Multi-scan (*CrysAlis PRO*; Rigaku OD, 2021 ▸)
*T*_min_, *T*_max_	0.805, 0.941
No. of measured, independent and observed [*I* > 2σ(*I*)] reflections	40427, 6866, 6251
*R*_int_	0.041
(sin θ/λ)_max_ (Å^−1^)	0.638

Refinement	
*R*[*F* ^2^ > 2σ(*F* ^2^)], *wR*(*F* ^2^), *S*	0.044, 0.114, 1.09
No. of reflections	6866
No. of parameters	411
No. of restraints	21
H-atom treatment	H atoms treated by a mixture of independent and constrained refinement
Δρ_max_, Δρ_min_ (e Å^−3^)	0.19, −0.20

Computer programs: *CrysAlis PRO* (Rigaku OD, 2021 ▸), *SHELXT* (Sheldrick, 2015*a* ▸), *SHELXL* (Sheldrick, 2015*b* ▸), *ORTEP-3 for Windows* (Farrugia, 2012 ▸) and *PLATON* (Spek, 2020 ▸).

## supplementary crystallographic information

### Crystal data

**Table:**

2C_16_H_27_NO·H_2_O	*F*(000) = 1144
*M**_r_* = 516.79	*D*_x_ = 1.085 Mg m^−^^3^
Monoclinic, *P*2_1_/*c*	Cu *K*α radiation, λ = 1.54184 Å
*a* = 13.06508 (16) Å	Cell parameters from 22446 reflections
*b* = 5.81242 (6) Å	θ = 2.2–79.1°
*c* = 41.7384 (5) Å	µ = 0.53 mm^−^^1^
β = 93.3315 (11)°	*T* = 100 K
*V* = 3164.25 (6) Å^3^	Plate, colourless
*Z* = 4	0.36 × 0.12 × 0.06 mm

### Data collection

**Table:**

XtaLAB Synergy, Dualflex, HyPix diffractometer	6251 reflections with *I* > 2σ(*I*)
Radiation source: micro-focus sealed X-ray tube	*R*_int_ = 0.041
φ and ω scans	θ_max_ = 79.7°, θ_min_ = 3.4°
Absorption correction: multi-scan (CrysAlisPro; Rigaku OD, 2021)	*h* = −16→16
*T*_min_ = 0.805, *T*_max_ = 0.941	*k* = −6→7
40427 measured reflections	*l* = −53→52
6866 independent reflections	

### Refinement

**Table:**

Refinement on *F*^2^	Primary atom site location: difference Fourier map
Least-squares matrix: full	Secondary atom site location: difference Fourier map
*R*[*F*^2^ > 2σ(*F*^2^)] = 0.044	Hydrogen site location: mixed
*wR*(*F*^2^) = 0.114	H atoms treated by a mixture of independent and constrained refinement
*S* = 1.09	*w* = 1/[σ^2^(*F*_o_^2^) + (0.0467*P*)^2^ + 0.9632*P*] where *P* = (*F*_o_^2^ + 2*F*_c_^2^)/3
6866 reflections	(Δ/σ)_max_ = 0.001
411 parameters	Δρ_max_ = 0.19 e Å^−^^3^
21 restraints	Δρ_min_ = −0.20 e Å^−^^3^

### Special details

**Table:**

Experimental. CrysAlisPro 1.171.41.117a (Rigaku OD, 2021) Empirical absorption correction using spherical harmonics, implemented in SCALE3 ABSPACK scaling algorithm.
Geometry. All esds (except the esd in the dihedral angle between two l.s. planes) are estimated using the full covariance matrix. The cell esds are taken into account individually in the estimation of esds in distances, angles and torsion angles; correlations between esds in cell parameters are only used when they are defined by crystal symmetry. An approximate (isotropic) treatment of cell esds is used for estimating esds involving l.s. planes.

### Fractional atomic coordinates and isotropic or equivalent isotropic displacement parameters (Å^2^)

**Table:**

	*x*	*y*	*z*	*U*_iso_*/*U*_eq_	Occ. (<1)
O1	0.83664 (7)	0.63481 (14)	0.38179 (2)	0.02938 (18)	
H1O	0.7755 (17)	0.562 (4)	0.3786 (5)	0.068 (6)*	
N1	0.75396 (7)	0.93228 (16)	0.42788 (2)	0.02387 (19)	
H1N	0.7600 (11)	0.774 (3)	0.4290 (3)	0.031 (4)*	
C1	0.82855 (8)	1.01229 (19)	0.40524 (3)	0.0256 (2)	
H1A	0.8989	0.9969	0.4152	0.031*	
H1B	0.8163	1.1769	0.4003	0.031*	
C2	0.81900 (8)	0.87226 (18)	0.37442 (3)	0.0252 (2)	
H2	0.7481	0.8905	0.3644	0.030*	
C3	0.89639 (9)	0.9587 (2)	0.35095 (3)	0.0293 (2)	
H3A	0.8870	1.1266	0.3481	0.035*	
H3B	0.9665	0.9334	0.3606	0.035*	
C4	0.88794 (9)	0.8449 (2)	0.31835 (3)	0.0291 (2)	
C5	0.81775 (9)	0.9285 (2)	0.29442 (3)	0.0325 (3)	
C6	0.81180 (9)	0.8239 (2)	0.26423 (3)	0.0360 (3)	
H6	0.7642	0.8817	0.2481	0.043*	
C7	0.87307 (10)	0.6384 (2)	0.25699 (3)	0.0363 (3)	
C8	0.94234 (10)	0.5581 (2)	0.28094 (3)	0.0359 (3)	
H8	0.9851	0.4314	0.2764	0.043*	
C9	0.95115 (9)	0.6570 (2)	0.31138 (3)	0.0322 (3)	
C10	0.74982 (11)	1.1329 (2)	0.30008 (3)	0.0393 (3)	
H10A	0.7097	1.1034	0.3188	0.059*	
H10B	0.7033	1.1580	0.2811	0.059*	
H10C	0.7924	1.2700	0.3040	0.059*	
C11	0.86603 (12)	0.5272 (3)	0.22430 (3)	0.0462 (3)	
H11A	0.8362	0.3732	0.2259	0.069*	
H11B	0.9348	0.5150	0.2162	0.069*	
H11C	0.8225	0.6210	0.2095	0.069*	
C12	1.03108 (10)	0.5649 (3)	0.33569 (3)	0.0400 (3)	
H12A	1.0620	0.4256	0.3272	0.060*	
H12B	0.9987	0.5279	0.3557	0.060*	
H12C	1.0843	0.6814	0.3400	0.060*	
C13	0.76617 (8)	1.02608 (19)	0.46103 (3)	0.0247 (2)	
C14	0.86787 (9)	0.9562 (2)	0.47850 (3)	0.0336 (3)	
H14A	0.8726	0.7880	0.4794	0.050*	
H14B	0.8710	1.0182	0.5004	0.050*	
H14C	0.9250	1.0176	0.4669	0.050*	
C15	0.67782 (9)	0.9273 (2)	0.47916 (3)	0.0288 (2)	
H15A	0.6125	0.9847	0.4695	0.043*	
H15B	0.6853	0.9748	0.5017	0.043*	
H15C	0.6790	0.7589	0.4779	0.043*	
C16	0.75688 (10)	1.2882 (2)	0.45967 (3)	0.0306 (2)	
H16A	0.8151	1.3522	0.4488	0.046*	
H16B	0.7568	1.3497	0.4815	0.046*	
H16C	0.6928	1.3306	0.4478	0.046*	
O2	0.57184 (6)	1.06462 (16)	0.39828 (2)	0.0352 (2)	
H2O	0.6278 (15)	1.004 (3)	0.4093 (5)	0.062 (5)*	
N2	0.50806 (8)	0.78162 (18)	0.34494 (2)	0.0306 (2)	
H2N	0.5598 (13)	0.887 (3)	0.3424 (4)	0.047 (4)*	
C17	0.43655 (8)	0.8875 (2)	0.36627 (3)	0.0283 (2)	
H17A	0.3746	0.7901	0.3671	0.034*	
H17B	0.4151	1.0399	0.3577	0.034*	
C18	0.48579 (8)	0.91565 (19)	0.39983 (3)	0.0264 (2)	
H18	0.5096	0.7620	0.4081	0.032*	
C19	0.41002 (9)	1.01744 (19)	0.42282 (3)	0.0271 (2)	
H19A	0.4465	1.0414	0.4440	0.032*	
H19B	0.3876	1.1702	0.4146	0.032*	
C20	0.31558 (8)	0.87209 (19)	0.42747 (2)	0.0244 (2)	
C21	0.32295 (8)	0.67813 (19)	0.44783 (2)	0.0246 (2)	
C22	0.23623 (9)	0.5454 (2)	0.45241 (3)	0.0269 (2)	
H22	0.2424	0.4139	0.4659	0.032*	
C23	0.14096 (9)	0.5996 (2)	0.43785 (3)	0.0286 (2)	
C24	0.13486 (8)	0.7904 (2)	0.41774 (3)	0.0287 (2)	
H24	0.0704	0.8298	0.4075	0.034*	
C25	0.21998 (9)	0.92618 (19)	0.41200 (3)	0.0264 (2)	
C26	0.42242 (9)	0.6101 (2)	0.46563 (3)	0.0288 (2)	
H26A	0.4727	0.5667	0.4502	0.043*	
H26B	0.4103	0.4793	0.4797	0.043*	
H26C	0.4488	0.7403	0.4785	0.043*	
C27	0.04768 (9)	0.4554 (2)	0.44353 (3)	0.0369 (3)	
H27A	0.0240	0.4888	0.4649	0.055*	
H27B	0.0654	0.2920	0.4422	0.055*	
H27C	−0.0070	0.4917	0.4272	0.055*	
C28	0.20503 (9)	1.1238 (2)	0.38851 (3)	0.0317 (2)	
H28A	0.2394	1.2613	0.3974	0.047*	
H28B	0.1316	1.1549	0.3847	0.047*	
H28C	0.2344	1.0828	0.3682	0.047*	
C29	0.46433 (9)	0.7090 (2)	0.31290 (3)	0.0363 (3)	
C30	0.5508 (4)	0.5955 (11)	0.29559 (17)	0.074 (2)	0.65 (3)
H30A	0.6091	0.7013	0.2952	0.111*	0.65 (3)
H30B	0.5266	0.5583	0.2735	0.111*	0.65 (3)
H30C	0.5722	0.4540	0.3069	0.111*	0.65 (3)
C31	0.3778 (4)	0.5356 (8)	0.31656 (15)	0.0441 (12)	0.65 (3)
H31A	0.3992	0.4212	0.3329	0.066*	0.65 (3)
H31B	0.3621	0.4583	0.2960	0.066*	0.65 (3)
H31C	0.3166	0.6162	0.3232	0.066*	0.65 (3)
C32	0.4236 (5)	0.9172 (8)	0.29385 (14)	0.0441 (12)	0.65 (3)
H32A	0.3700	0.9927	0.3055	0.066*	0.65 (3)
H32B	0.3950	0.8668	0.2728	0.066*	0.65 (3)
H32C	0.4797	1.0258	0.2910	0.066*	0.65 (3)
C30'	0.5561 (7)	0.632 (3)	0.2945 (2)	0.071 (4)	0.35 (3)
H30D	0.5967	0.7664	0.2891	0.107*	0.35 (3)
H30E	0.5317	0.5529	0.2748	0.107*	0.35 (3)
H30F	0.5987	0.5265	0.3079	0.107*	0.35 (3)
C31'	0.3957 (13)	0.503 (2)	0.3189 (3)	0.088 (5)	0.35 (3)
H31D	0.4373	0.3773	0.3285	0.132*	0.35 (3)
H31E	0.3625	0.4508	0.2985	0.132*	0.35 (3)
H31F	0.3432	0.5472	0.3336	0.132*	0.35 (3)
C32'	0.4036 (12)	0.891 (2)	0.2936 (3)	0.044 (2)	0.35 (3)
H32D	0.3457	0.9426	0.3057	0.066*	0.35 (3)
H32E	0.3779	0.8259	0.2731	0.066*	0.35 (3)
H32F	0.4482	1.0228	0.2897	0.066*	0.35 (3)
O3	0.6489 (2)	0.4428 (10)	0.3681 (3)	0.0388 (14)	0.59 (3)
O3'	0.6420 (5)	0.4819 (13)	0.3828 (5)	0.044 (3)	0.41 (3)
H3C	0.6156 (16)	0.335 (4)	0.3812 (5)	0.072 (6)*	
H3D	0.5940 (18)	0.556 (4)	0.3639 (6)	0.083 (7)*	

### Atomic displacement parameters (Å^2^)

**Table:**

	*U*^11^	*U*^22^	*U*^33^	*U*^12^	*U*^13^	*U*^23^
O1	0.0295 (4)	0.0246 (4)	0.0344 (4)	0.0009 (3)	0.0046 (3)	0.0014 (3)
N1	0.0240 (4)	0.0230 (4)	0.0249 (4)	−0.0032 (3)	0.0039 (3)	−0.0005 (3)
C1	0.0234 (5)	0.0268 (5)	0.0270 (5)	−0.0030 (4)	0.0039 (4)	0.0010 (4)
C2	0.0232 (5)	0.0250 (5)	0.0277 (5)	0.0005 (4)	0.0038 (4)	0.0019 (4)
C3	0.0270 (5)	0.0315 (6)	0.0300 (6)	−0.0026 (4)	0.0063 (4)	0.0010 (5)
C4	0.0259 (5)	0.0331 (6)	0.0290 (5)	−0.0024 (4)	0.0086 (4)	0.0021 (4)
C5	0.0287 (6)	0.0386 (6)	0.0312 (6)	0.0008 (5)	0.0095 (4)	0.0042 (5)
C6	0.0307 (6)	0.0487 (7)	0.0291 (6)	0.0007 (5)	0.0058 (5)	0.0030 (5)
C7	0.0313 (6)	0.0458 (7)	0.0327 (6)	−0.0048 (5)	0.0099 (5)	−0.0035 (5)
C8	0.0301 (6)	0.0398 (7)	0.0388 (6)	0.0019 (5)	0.0105 (5)	−0.0044 (5)
C9	0.0257 (5)	0.0375 (6)	0.0342 (6)	0.0003 (5)	0.0081 (4)	0.0004 (5)
C10	0.0409 (7)	0.0437 (7)	0.0341 (6)	0.0098 (6)	0.0088 (5)	0.0077 (5)
C11	0.0438 (7)	0.0593 (9)	0.0363 (7)	−0.0035 (7)	0.0082 (6)	−0.0105 (6)
C12	0.0322 (6)	0.0471 (7)	0.0408 (7)	0.0089 (5)	0.0036 (5)	−0.0019 (6)
C13	0.0251 (5)	0.0251 (5)	0.0240 (5)	−0.0023 (4)	0.0009 (4)	0.0000 (4)
C14	0.0292 (6)	0.0405 (6)	0.0305 (6)	−0.0014 (5)	−0.0024 (4)	0.0030 (5)
C15	0.0311 (6)	0.0297 (5)	0.0260 (5)	−0.0032 (4)	0.0046 (4)	0.0002 (4)
C16	0.0377 (6)	0.0258 (5)	0.0285 (5)	−0.0035 (5)	0.0028 (5)	−0.0026 (4)
O2	0.0249 (4)	0.0390 (5)	0.0412 (5)	−0.0096 (3)	−0.0023 (3)	0.0157 (4)
N2	0.0274 (5)	0.0342 (5)	0.0307 (5)	0.0032 (4)	0.0048 (4)	0.0018 (4)
C17	0.0247 (5)	0.0339 (6)	0.0266 (5)	0.0012 (4)	0.0028 (4)	0.0027 (4)
C18	0.0229 (5)	0.0273 (5)	0.0288 (5)	−0.0046 (4)	0.0006 (4)	0.0058 (4)
C19	0.0286 (5)	0.0265 (5)	0.0258 (5)	−0.0043 (4)	−0.0010 (4)	0.0008 (4)
C20	0.0252 (5)	0.0267 (5)	0.0215 (5)	−0.0001 (4)	0.0027 (4)	−0.0021 (4)
C21	0.0240 (5)	0.0284 (5)	0.0215 (5)	−0.0006 (4)	0.0025 (4)	−0.0016 (4)
C22	0.0275 (5)	0.0298 (5)	0.0236 (5)	−0.0016 (4)	0.0037 (4)	0.0012 (4)
C23	0.0240 (5)	0.0356 (6)	0.0266 (5)	−0.0022 (4)	0.0045 (4)	−0.0027 (4)
C24	0.0221 (5)	0.0373 (6)	0.0268 (5)	0.0034 (4)	0.0013 (4)	−0.0024 (5)
C25	0.0279 (5)	0.0288 (5)	0.0227 (5)	0.0029 (4)	0.0024 (4)	−0.0023 (4)
C26	0.0263 (5)	0.0319 (6)	0.0278 (5)	−0.0016 (4)	−0.0005 (4)	0.0050 (4)
C27	0.0267 (6)	0.0455 (7)	0.0389 (6)	−0.0052 (5)	0.0048 (5)	0.0025 (5)
C28	0.0305 (6)	0.0341 (6)	0.0303 (6)	0.0058 (5)	0.0007 (4)	0.0033 (5)
C29	0.0402 (7)	0.0371 (6)	0.0319 (6)	0.0003 (5)	0.0058 (5)	−0.0048 (5)
C30	0.049 (3)	0.105 (4)	0.071 (4)	0.004 (3)	0.024 (3)	−0.038 (3)
C31	0.055 (2)	0.0376 (16)	0.0394 (19)	−0.0078 (15)	0.0027 (13)	−0.0046 (13)
C32	0.055 (2)	0.052 (2)	0.0242 (16)	−0.0116 (16)	−0.0053 (14)	0.0028 (14)
C30'	0.087 (8)	0.087 (6)	0.038 (5)	0.049 (5)	−0.008 (4)	−0.029 (4)
C31'	0.147 (11)	0.053 (5)	0.060 (5)	−0.052 (6)	−0.023 (7)	−0.003 (4)
C32'	0.044 (4)	0.044 (3)	0.041 (4)	−0.004 (3)	−0.012 (3)	−0.005 (3)
O3	0.0282 (9)	0.0331 (13)	0.055 (3)	−0.0023 (7)	0.0027 (11)	0.0153 (17)
O3'	0.0310 (15)	0.0302 (17)	0.068 (7)	−0.0053 (12)	−0.011 (2)	0.016 (3)

### Geometric parameters (Å, º)

**Table:**

O1—C2	1.4298 (13)	C18—C19	1.5367 (16)
O1—H1O	0.91 (2)	C18—H18	1.0000
N1—C1	1.4718 (13)	C19—C20	1.5171 (15)
N1—C13	1.4872 (14)	C19—H19A	0.9900
N1—H1N	0.922 (16)	C19—H19B	0.9900
C1—C2	1.5211 (15)	C20—C25	1.4079 (15)
C1—H1A	0.9900	C20—C21	1.4118 (15)
C1—H1B	0.9900	C21—C22	1.3930 (15)
C2—C3	1.5330 (15)	C21—C26	1.5115 (15)
C2—H2	1.0000	C22—C23	1.3892 (16)
C3—C4	1.5117 (16)	C22—H22	0.9500
C3—H3A	0.9900	C23—C24	1.3899 (17)
C3—H3B	0.9900	C23—C27	1.5094 (16)
C4—C5	1.4030 (17)	C24—C25	1.3958 (16)
C4—C9	1.4096 (17)	C24—H24	0.9500
C5—C6	1.3972 (18)	C25—C28	1.5151 (16)
C5—C10	1.5097 (18)	C26—H26A	0.9800
C6—C7	1.3863 (19)	C26—H26B	0.9800
C6—H6	0.9500	C26—H26C	0.9800
C7—C8	1.3896 (19)	C27—H27A	0.9800
C7—C11	1.5081 (18)	C27—H27B	0.9800
C8—C9	1.3930 (18)	C27—H27C	0.9800
C8—H8	0.9500	C28—H28A	0.9800
C9—C12	1.5112 (18)	C28—H28B	0.9800
C10—H10A	0.9800	C28—H28C	0.9800
C10—H10B	0.9800	C29—C32'	1.525 (3)
C10—H10C	0.9800	C29—C30	1.526 (2)
C11—H11A	0.9800	C29—C31'	1.527 (3)
C11—H11B	0.9800	C29—C32	1.527 (2)
C11—H11C	0.9800	C29—C30'	1.528 (3)
C12—H12A	0.9800	C29—C31	1.529 (2)
C12—H12B	0.9800	C30—H30A	0.9800
C12—H12C	0.9800	C30—H30B	0.9800
C13—C15	1.5284 (15)	C30—H30C	0.9800
C13—C16	1.5289 (16)	C31—H31A	0.9800
C13—C14	1.5328 (15)	C31—H31B	0.9800
C14—H14A	0.9800	C31—H31C	0.9800
C14—H14B	0.9800	C32—H32A	0.9800
C14—H14C	0.9800	C32—H32B	0.9800
C15—H15A	0.9800	C32—H32C	0.9800
C15—H15B	0.9800	C30'—H30D	0.9800
C15—H15C	0.9800	C30'—H30E	0.9800
C16—H16A	0.9800	C30'—H30F	0.9800
C16—H16B	0.9800	C31'—H31D	0.9800
C16—H16C	0.9800	C31'—H31E	0.9800
O2—C18	1.4235 (13)	C31'—H31F	0.9800
O2—H2O	0.91 (2)	C32'—H32D	0.9800
N2—C17	1.4631 (15)	C32'—H32E	0.9800
N2—C29	1.4846 (16)	C32'—H32F	0.9800
N2—H2N	0.922 (18)	O3—H3C	0.95 (2)
C17—C18	1.5158 (16)	O3—H3D	0.98 (3)
C17—H17A	0.9900	O3'—H3C	0.92 (2)
C17—H17B	0.9900	O3'—H3D	1.07 (3)
			
C2—O1—H1O	106.9 (13)	O2—C18—H18	109.1
C1—N1—C13	116.25 (8)	C17—C18—H18	109.1
C1—N1—H1N	106.8 (9)	C19—C18—H18	109.1
C13—N1—H1N	108.2 (9)	C20—C19—C18	115.06 (9)
N1—C1—C2	110.42 (9)	C20—C19—H19A	108.5
N1—C1—H1A	109.6	C18—C19—H19A	108.5
C2—C1—H1A	109.6	C20—C19—H19B	108.5
N1—C1—H1B	109.6	C18—C19—H19B	108.5
C2—C1—H1B	109.6	H19A—C19—H19B	107.5
H1A—C1—H1B	108.1	C25—C20—C21	118.82 (10)
O1—C2—C1	109.29 (9)	C25—C20—C19	121.57 (10)
O1—C2—C3	110.48 (9)	C21—C20—C19	119.61 (10)
C1—C2—C3	109.88 (9)	C22—C21—C20	119.82 (10)
O1—C2—H2	109.1	C22—C21—C26	118.08 (10)
C1—C2—H2	109.1	C20—C21—C26	122.09 (10)
C3—C2—H2	109.1	C23—C22—C21	121.98 (11)
C4—C3—C2	114.54 (9)	C23—C22—H22	119.0
C4—C3—H3A	108.6	C21—C22—H22	119.0
C2—C3—H3A	108.6	C22—C23—C24	117.61 (10)
C4—C3—H3B	108.6	C22—C23—C27	121.08 (11)
C2—C3—H3B	108.6	C24—C23—C27	121.30 (11)
H3A—C3—H3B	107.6	C23—C24—C25	122.45 (10)
C5—C4—C9	119.21 (11)	C23—C24—H24	118.8
C5—C4—C3	119.98 (11)	C25—C24—H24	118.8
C9—C4—C3	120.79 (11)	C24—C25—C20	119.30 (10)
C6—C5—C4	119.40 (11)	C24—C25—C28	117.76 (10)
C6—C5—C10	118.77 (11)	C20—C25—C28	122.93 (10)
C4—C5—C10	121.81 (11)	C21—C26—H26A	109.5
C7—C6—C5	122.16 (12)	C21—C26—H26B	109.5
C7—C6—H6	118.9	H26A—C26—H26B	109.5
C5—C6—H6	118.9	C21—C26—H26C	109.5
C6—C7—C8	117.70 (12)	H26A—C26—H26C	109.5
C6—C7—C11	121.66 (12)	H26B—C26—H26C	109.5
C8—C7—C11	120.64 (12)	C23—C27—H27A	109.5
C7—C8—C9	122.21 (12)	C23—C27—H27B	109.5
C7—C8—H8	118.9	H27A—C27—H27B	109.5
C9—C8—H8	118.9	C23—C27—H27C	109.5
C8—C9—C4	119.31 (11)	H27A—C27—H27C	109.5
C8—C9—C12	118.91 (11)	H27B—C27—H27C	109.5
C4—C9—C12	121.74 (11)	C25—C28—H28A	109.5
C5—C10—H10A	109.5	C25—C28—H28B	109.5
C5—C10—H10B	109.5	H28A—C28—H28B	109.5
H10A—C10—H10B	109.5	C25—C28—H28C	109.5
C5—C10—H10C	109.5	H28A—C28—H28C	109.5
H10A—C10—H10C	109.5	H28B—C28—H28C	109.5
H10B—C10—H10C	109.5	N2—C29—C32'	116.0 (6)
C7—C11—H11A	109.5	N2—C29—C30	107.0 (3)
C7—C11—H11B	109.5	N2—C29—C31'	106.0 (6)
H11A—C11—H11B	109.5	C32'—C29—C31'	109.9 (3)
C7—C11—H11C	109.5	N2—C29—C32	110.3 (3)
H11A—C11—H11C	109.5	C30—C29—C32	109.9 (2)
H11B—C11—H11C	109.5	N2—C29—C30'	105.3 (5)
C9—C12—H12A	109.5	C32'—C29—C30'	109.8 (3)
C9—C12—H12B	109.5	C31'—C29—C30'	109.7 (3)
H12A—C12—H12B	109.5	N2—C29—C31	110.2 (3)
C9—C12—H12C	109.5	C30—C29—C31	109.6 (2)
H12A—C12—H12C	109.5	C32—C29—C31	109.7 (2)
H12B—C12—H12C	109.5	C29—C30—H30A	109.5
N1—C13—C15	106.23 (8)	C29—C30—H30B	109.5
N1—C13—C16	109.07 (9)	H30A—C30—H30B	109.5
C15—C13—C16	109.40 (9)	C29—C30—H30C	109.5
N1—C13—C14	112.86 (9)	H30A—C30—H30C	109.5
C15—C13—C14	108.89 (9)	H30B—C30—H30C	109.5
C16—C13—C14	110.27 (10)	C29—C31—H31A	109.5
C13—C14—H14A	109.5	C29—C31—H31B	109.5
C13—C14—H14B	109.5	H31A—C31—H31B	109.5
H14A—C14—H14B	109.5	C29—C31—H31C	109.5
C13—C14—H14C	109.5	H31A—C31—H31C	109.5
H14A—C14—H14C	109.5	H31B—C31—H31C	109.5
H14B—C14—H14C	109.5	C29—C32—H32A	109.5
C13—C15—H15A	109.5	C29—C32—H32B	109.5
C13—C15—H15B	109.5	H32A—C32—H32B	109.5
H15A—C15—H15B	109.5	C29—C32—H32C	109.5
C13—C15—H15C	109.5	H32A—C32—H32C	109.5
H15A—C15—H15C	109.5	H32B—C32—H32C	109.5
H15B—C15—H15C	109.5	C29—C30'—H30D	109.5
C13—C16—H16A	109.5	C29—C30'—H30E	109.5
C13—C16—H16B	109.5	H30D—C30'—H30E	109.5
H16A—C16—H16B	109.5	C29—C30'—H30F	109.5
C13—C16—H16C	109.5	H30D—C30'—H30F	109.5
H16A—C16—H16C	109.5	H30E—C30'—H30F	109.5
H16B—C16—H16C	109.5	C29—C31'—H31D	109.5
C18—O2—H2O	110.6 (13)	C29—C31'—H31E	109.5
C17—N2—C29	116.22 (9)	H31D—C31'—H31E	109.5
C17—N2—H2N	106.8 (11)	C29—C31'—H31F	109.5
C29—N2—H2N	109.4 (11)	H31D—C31'—H31F	109.5
N2—C17—C18	110.86 (9)	H31E—C31'—H31F	109.5
N2—C17—H17A	109.5	C29—C32'—H32D	109.5
C18—C17—H17A	109.5	C29—C32'—H32E	109.5
N2—C17—H17B	109.5	H32D—C32'—H32E	109.5
C18—C17—H17B	109.5	C29—C32'—H32F	109.5
H17A—C17—H17B	108.1	H32D—C32'—H32F	109.5
O2—C18—C17	108.52 (9)	H32E—C32'—H32F	109.5
O2—C18—C19	109.47 (9)	H3C—O3—H3D	100.9 (19)
C17—C18—C19	111.38 (9)	H3C—O3'—H3D	97 (2)
			
C13—N1—C1—C2	169.98 (9)	N2—C17—C18—C19	−177.64 (9)
N1—C1—C2—O1	−59.13 (11)	O2—C18—C19—C20	−178.38 (9)
N1—C1—C2—C3	179.49 (9)	C17—C18—C19—C20	61.61 (12)
O1—C2—C3—C4	64.12 (12)	C18—C19—C20—C25	−102.29 (12)
C1—C2—C3—C4	−175.21 (10)	C18—C19—C20—C21	77.93 (13)
C2—C3—C4—C5	85.11 (13)	C25—C20—C21—C22	−0.39 (16)
C2—C3—C4—C9	−95.98 (13)	C19—C20—C21—C22	179.39 (10)
C9—C4—C5—C6	0.10 (17)	C25—C20—C21—C26	−179.24 (10)
C3—C4—C5—C6	179.03 (11)	C19—C20—C21—C26	0.55 (16)
C9—C4—C5—C10	−178.41 (11)	C20—C21—C22—C23	−1.02 (17)
C3—C4—C5—C10	0.52 (17)	C26—C21—C22—C23	177.87 (10)
C4—C5—C6—C7	0.16 (19)	C21—C22—C23—C24	1.29 (17)
C10—C5—C6—C7	178.71 (12)	C21—C22—C23—C27	−179.10 (11)
C5—C6—C7—C8	−0.30 (19)	C22—C23—C24—C25	−0.17 (17)
C5—C6—C7—C11	−179.92 (12)	C27—C23—C24—C25	−179.78 (11)
C6—C7—C8—C9	0.20 (19)	C23—C24—C25—C20	−1.20 (17)
C11—C7—C8—C9	179.81 (12)	C23—C24—C25—C28	177.42 (11)
C7—C8—C9—C4	0.05 (19)	C21—C20—C25—C24	1.46 (16)
C7—C8—C9—C12	−177.80 (12)	C19—C20—C25—C24	−178.32 (10)
C5—C4—C9—C8	−0.20 (17)	C21—C20—C25—C28	−177.09 (10)
C3—C4—C9—C8	−179.12 (11)	C19—C20—C25—C28	3.13 (16)
C5—C4—C9—C12	177.59 (11)	C17—N2—C29—C32'	52.0 (7)
C3—C4—C9—C12	−1.33 (17)	C17—N2—C29—C30	−177.7 (3)
C1—N1—C13—C15	177.41 (9)	C17—N2—C29—C31'	−70.2 (8)
C1—N1—C13—C16	59.59 (12)	C17—N2—C29—C32	62.7 (3)
C1—N1—C13—C14	−63.33 (12)	C17—N2—C29—C30'	173.6 (6)
C29—N2—C17—C18	170.22 (10)	C17—N2—C29—C31	−58.6 (3)
N2—C17—C18—O2	61.79 (12)		

### Hydrogen-bond geometry (Å, º)

*Cg*2 is the centroid of the benzene ring (C4–C9) of molecule *A*.

**Table:**

*D*—H···*A*	*D*—H	H···*A*	*D*···*A*	*D*—H···*A*
O1—H1*O*···O3	0.91 (2)	1.82 (2)	2.725 (5)	173 (2)
O1—H1*O*···O3′	0.91 (2)	1.82 (2)	2.697 (6)	161 (2)
O2—H2*O*···N1	0.91 (2)	1.83 (2)	2.7273 (13)	168.0 (19)
O3—H3*C*···O2^i^	0.95 (2)	1.83 (2)	2.753 (3)	162 (2)
O3′—H3*C*···O2^i^	0.92 (2)	1.83 (2)	2.685 (4)	153 (2)
O3—H3*D*···N2	0.98 (3)	1.87 (3)	2.827 (3)	164 (2)
O3′—H3*D*···N2	1.07 (3)	1.87 (3)	2.875 (5)	155 (2)
C11—H11*B*···*Cg*2^ii^	0.98	2.90	3.7613 (17)	147

Symmetry codes: (i) *x*, *y*−1, *z*; (ii) −*x*+2, *y*−1/2, −*z*+1/2.
